# Identification of a Prognostic Signature for Ovarian Cancer Based on the Microenvironment Genes

**DOI:** 10.3389/fgene.2021.680413

**Published:** 2021-05-13

**Authors:** Xiao Huo, Hengzi Sun, Shuangwu Liu, Bing Liang, Huimin Bai, Shuzhen Wang, Shuhong Li

**Affiliations:** ^1^Peking University Third Hospital Institute of Medical Innovation and Research, Beijing, China; ^2^Department of Obstetrics and Gynecology, Beijing Chao-Yang Hospital, Capital Medical University, Beijing, China; ^3^School of Medicine, ShanDong University, Jinan, China

**Keywords:** ovarian cancer, microenvironment, immune metagenes, prognosis, TCGA

## Abstract

**Background**: Ovarian cancer is highly malignant and has a poor prognosis in the advanced stage. Studies have shown that infiltration of tumor microenvironment cells, immune cells and stromal cells has an important impact on the prognosis of cancers. However, the relationship between tumor microenvironment genes and the prognosis of ovarian cancer has not been studied.

**Methods**: Gene expression profiles and SNP data of ovarian cancer were downloaded from the TCGA database. Cluster analysis, WGCNA analysis and univariate survival analysis were used to identify immune microenvironment genes as prognostic signatures for predicting the survival of ovarian cancer patients. External data were used to evaluate the signature. Moreover, the top five significantly correlated genes were evaluated by immunohistochemical staining of ovarian cancer tissues.

**Results**: We systematically analyzed the relationship between ovarian cancer and immune metagenes. Immune metagenes expression were associated with prognosis. In total, we identified 10 genes related to both immunity and prognosis in ovarian cancer according to the expression of immune metagenes. These data reveal that high expression of ETV7 (OS, HR = 1.540, 95% CI 1.023–2.390, *p* = 0.041), GBP4 (OS, HR = 1.834, 95% CI 1.242–3.055, *p* = 0.004), CXCL9 (OS, HR = 1.613, 95% CI 1.080 –2.471, *p* = 0.021), CD3E (OS, HR = 1.590, 95% CI 1.049 –2.459, *p* = 0.031), and TAP1 (OS, HR = 1.766, 95% CI 1.163 –2.723, *p* = 0.009) are associated with better prognosis in patients with ovarian cancer.

**Conclusion**: Our study identified 10 immune microenvironment genes related to the prognosis of ovarian cancer. The list of tumor microenvironment-related genes provides new insights into the underlying biological mechanisms driving the tumorigenesis of ovarian cancer.

## Introduction

Cancer seriously endangers human health, and in recent years, the incidence of malignant tumors has increased annually. The World Health Organization reported 18.1 million new cancer cases and 9.6 million cancer-related deaths worldwide in 2018. Ovarian cancer is a common gynecologic malignancy and the fifth leading cause of cancer-related deaths in women (Siegel et al., 2018). The lifetime risk of ovarian cancer in women is 1.3%. The 5-year survival rate ranged from 29 to 93%, depending on the initial diagnosis (Torre et al., 2018). Despite advances in treatment strategies and techniques, the mortality rate of ovarian cancer remains high. The main reason is the lack of obvious symptoms and effective screening for ovarian cancer. Sixty percent of patients were diagnosed with advanced ovarian cancer (Dinh et al., 2008). Standard treatment for advanced ovarian cancer includes tumor cell destruction and standard chemotherapy. However, most patients relapse within 2–3 years after first-line chemotherapy and die as a consequence of chemotherapy resistance (Odunsi, 2017). Thus, new treatment strategies and paradigms are greatly needed for these patients.

Malignant solid tumor tissue is heterogeneous and includes not only tumor cells but also tumor-associated normal epithelial and stromal cells, immune cells and vascular cells. The process of tumor development depends on a variety of complex signaling pathways between tumor cells and the tumor microenvironment (Kreuzinger et al., 2017). With the improvement of understanding the molecular basis of immune recognition and immune regulation in tumor cells, immunotherapy has aroused great interest (Nelson, 2015). Tumor microenvironment cells and the degree of infiltration of immune and stromal cells in tumors have been reported to significantly contribute to the prognosis. In the tumor microenvironment, immune and stromal cells are two main types of non-tumor components and have been proposed to be valuable for the diagnosis and prognosis evaluations of tumors (Senbabaoglu et al., 2016; Winslow et al., 2016; Ovarian Tumor Tissue Analysis (Otta) Consortium, Goode et al., 2017). Many algorithms have been developed to calculate tumor purity using gene expression and DNA methylation data (Carter et al., 2012; Yoshihara et al., 2013; Zheng et al., 2017). The immune and stromal scores calculated based on the ESTIMATE algorithm (Yoshihara et al., 2013) promote the quantitative determination of immune and stromal components in tumors. In this algorithm, the authors calculated immune and stromal scores by analyzing specific gene expression characteristics of immune and stromal cells to predict non-tumor cell infiltration. This algorithm has been applied to prostate cancer (Shah et al., 2017) and breast cancer (Jia et al., 2018), and the results show the effectiveness of this algorithm, but there are no detailed studies on ovarian cancer.

The Cancer Genome Atlas (TCGA) has been established to improve cancer prevention, diagnosis and treatment by applying high-throughput genome analysis techniques to provide a better understanding of cancer (Cancer Genome Atlas Research Network, 2008). To better understand the effect of immune microenvironment-related genes on the prognosis of ovarian cancer, we systematically analyzed the expression profile data in the TCGA database and mined the genes related to the microenvironment of ovarian cancer and poor prognosis. Finally, we obtained a set of microenvironment genes associated with poor prognosis in ovarian cancer patients and validated them with the online tool KMplot^1^.

## Materials and Methods

### Data Source and Data Pre-processing

#### TCGA Data

We used the GDC API to download level 3 data for OC patients from the TCGA database^2^ (December 26, 2018). The data included the following: (1) RNA-seq data (*n* = 379). The Fragment Per Kilobase of transcript per Million mapped reads (FPKM) data of RNA-Seq were downloaded from the TCGA and further converted into Transcript Per Million (TPM) expression profiles and RNA-Seq Count data; (2) Single nucleotide polymorphism (SNP) data (*n* = 436); and (3) Clinical follow-up information (*n* = 587) including survival and outcome.

#### Immune Metagenes Scores

Thirteen kinds of immune metagenes, which correspond to various types of immune cells and reflect various immune functions, were identified from previous reports (Safonov et al., 2017). For each sample, according to the gene expression levels of immune metagenes, we selected the median expression level of each type of immune metagenes and designated these levels as the immune metagenes score for these samples.

#### Immune Cell Scores

We downloaded the scores of six types of immune cells corresponding to each sample of ovarian cancer from the Tumor Immune Estimation Resource (TIMER)^3^ database. The six types of immune cells were B cells, CD4^+^ T cells, CD8^+^ T cells, neutrophils, macrophages and dendritic cells.

#### Immune Scores and Stromal Scores

Stromal and immune scores were estimated from transcriptomic profiles of the ovarian cancer cohort from TCGA using the ESTIMATE algorithm. We used the R software package estimate to calculate the immune and stromal scores of each sample. ESTIMATE (Estimation of STromal and Immune cells in MAlignant Tumor tissues using Expression data) is a tool for predicting tumor purity, and the presence of infiltrating stromal/immune cells in tumor tissues using gene expression data. ESTIMATE algorithm is based on single sample Gene Set Enrichment Analysis and generates three scores: stromal score (that captures the presence of stroma in tumor tissue), immune score (that represents the infiltration of immune cells in tumor tissue), and estimate score (that infers tumor purity).

### Overall Survival Curve and Differential Expression Analysis

The data were processed: (1) KM plots were generated to illustrate the relationship between patients’ overall survival and gene expression levels of immune metagenes. The relationship was tested by log-rank test. (2) Weighted gene co-expression network analysis (WGCNA), an R software package (Langfelder and Horvath, 2008; Wang et al., 2019), was used to construct a weighted co-expression network. A soft threshold of 8 was selected to screen the co-expression modules. The protein-protein interaction (PPI) network was retrieved from STRING database (Szklarczyk et al., 2015) and reconstructed via Cytoscape software (Shannon et al., 2003; Wang et al., 2020). (3) The R software package clusterProfiler for KEGG enrichment analysis was used, and a significance of false discover rate (FDR) < 0.05 was selected. (4) Data analysis was performed using the package DESeq2. The log2 (Foldchange)| > 1 and FDR < 0.05 were set as the cutoff values to screen for differentially expressed genes.

### Immunohistochemical Staining (IHC)

We collected a total of 168 human ovarian cancer tissue samples, which had accompanying follow-up information, from archives of paraffin-embedded tissues between January 2010 and January 2015 at the Department of Pathology of Beijing Chao-Yang Hospital. The follow-up was performed until December 31, 2020. The pathological diagnoses were reconfirmed by a pathologist. The patients included in present study were all (1) Epithelial ovarian cancer, (2) Underwent cytoreductive surgery and subsequent chemotherapy, (3) With follow-up information. The exclusion criteria were (1) Ovarian germ cell tumor, ovarian sex cord stromal tumor or metastatic cancer, (2) Unstandardized treatment, (3) No informed consent, (4) Lost to follow-up, and (5) No enough pathological samples.

The project was approved by the Ethical Committee (Beijing Chao-Yang Hospital), and informed consent was acquired from patients. IHC was performed as previously described (Li et al., 2010). Antibodies against the following were used: ETV7 1:200 abcam ab229832, GBP4 1:50 abcam ab232693, CXCL9 1:100 abcam ab137792, CD3E 1:500 abcam ab237721, TAP1 1:200 abcam ab137013. The scoring details have been described previously (Zhang et al., 2015). The intensity of immunostaining was graded as follows: 1+, weak; 2+, moderate; 3+, strong or 4+, very strong. The area of positive cancer cells in each microscopic field was categorized as follows: 1+, 0–25%; 2+, 25–50%; 3+, 50–75%, or 4+, 75–100%. The sum between 5 and 80 was obtained by multiplying the two scores by 5. A sum from 0 to 42 was assigned as “low expression” and that from 43 to 80 as “high expression.” All pathological diagnoses were confirmed in a blinded manner by three expert pathologists.

## Results

### Correlation Analysis of Immune Metagenes With Immunological Components in the Tumor Microenvironment

To observe the relationship between 13 types of immune metagenes scores, we calculated the correlation between them, as shown in Supplementary Figure 1A. The average correlations of natural killer cells (NK), regulatory T cells (Tregs), interferon-inducible genes (IF_I) and major histocompatibility complex class II antigen (MHC2) with other metagenes were the smallest and were 0.08157227, 0.23253018, 0.3120958, and 0.398014, respectively. The other classes of metagenes were highly correlated, which indicates that there is a certain consistency in the expression of metagenes in ovarian cancer. Furthermore, we analyzed the immune metagenes scores and six kinds of immune cells in the tumor microenvironment, as shown in Supplementary Figure 1B. We found that in addition to NK, Tregs and IF_I have smaller correlations with the content of six kinds of immune cells, and the scores for other metagenes were >0.4, suggesting that the immune metagenes and immune cells in the immune microenvironment have a significant correlation. Finally, we calculated the correlation between immune metagenes and immune and stromal scores, as shown in Supplementary Figure 1C. The correlation of the other 10 types of immune metagenes, except for NK, IF_I and Tregs, was very high, with an average higher than 0.4. In conclusion, the expression of these immune metagenes was closely related to the immune components in the tumor microenvironment.

### Relationship Between Immune Metagenes and Clinical Stage

According to the expression levels and stages of immune metagenes in each sample, we calculated the expression level distribution of immune metagenes in different stages, as shown in Supplementary Figures 2A–M (the number of Stage I samples was too small to be counted, so we counted only Stages II-IV). Immune metagenes showed a trend of successively declining expression of Stages II-IV, and ImmuneScore, follicular helper T cells (Tfh) and signal transducer and activator of transcription 1 (STAT1) had significant differences in various stages. The prognostic differences in the four stages were further analyzed as shown in Supplementary Figure 2N, and different stages had significant prognostic differences. This result suggests that the expression of immune metagenes may be closely related to the prognosis of ovarian cancer.

### Prognostic Difference Analysis of Immune Metagenes

**FIGURE 1:**
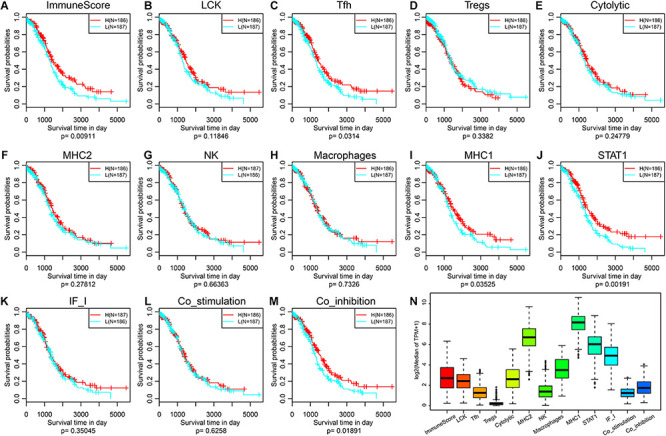
**(A–M)** KM curve of the expression and prognosis of immune metagenes; group H represents high-expression genes, and group L represents low-expression genes. **(N)** The expression distribution of immune metagenes.

To observe the expression and prognosis of the relationship between immune metagenes, we classified as high- and low-expression samples according to the median expression of metagenes. KM plots was used for prognostic difference analysis, as shown in Figures 1A–M. In all immune metagenes, the low-expression group had a worse prognosis than the high-expression group, in which ImmuneScore, Tfh, MHC1, STAT1 and Co_inhibition showed significant differences in prognosis, suggesting that the high expression of metagenes was a good prognostic factor. Next, we analyzed the expression distribution of immune metagenes as shown in Figure 1N. Except for the low expression of Tregs, the median expression of other types of metagenes was generally high. This result suggests that these immune metagenes are commonly expressed genes in ovarian cancer, indicating the potential of these metagenes as a new prognostic marker.

### Relationship Between Immune Metagenes and BRCA Mutations

BRCA genes are tumor suppressor genes that play important roles in cell replication regulation, DNA damage repair and normal cell growth. If BRCA genes are mutated, they will lose their ability to inhibit tumorigenesis. There are hundreds of BRCA mutation types, which are related to the occurrence of many cancers in the human body; among these cancers, breast cancer is the most closely related to *BRCA* mutations, followed by ovarian cancer. Therefore, we analyzed the relationship between these immune metagenes and BRCA1 and BRCA2 mutations. First, MuTect (Cibulskis et al., 2013) was used to process SNP data downloaded from the TCGA and to extract mutation data of BRCA1 and BRCA2. The expression relationship of immune metagenes in the BRCA1 mutation group and wild-type group samples was analyzed as shown in Supplementary Figures 3A–M. There were eight immune metagenes with significant expression differences, and the expression of the wild-type group was significantly higher than the mutant group. In addition, Macrophages, MHC1 and STAT1 had no significant differences, but the *P*-value was on the edge of significance. Second, we analyzed the differences in expression for immune metagenes between the BRCA2 mutation group and the wild-type group, as shown in Supplementary Figures 3N–Z. There were no significant differences in metagene expression among immune metagenes. This finding is consistent with previous studies and shows that BRCA2 mutations in ovarian cancer have no prognostic significance (Goode et al., 2017).

### WGCNA Analysis Mining Immune Metagenes Related Modules

**FIGURE 2:**
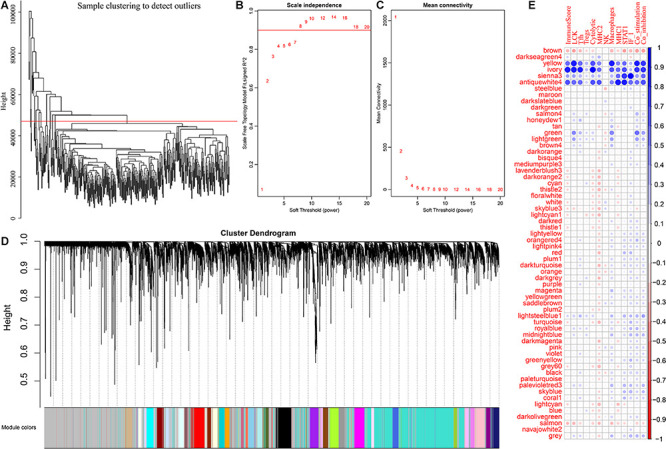
**(A)** The sample clustering analysis. **(B,C)** Analysis of network topology for various soft-thresholding powers. **(D)** The figure shows gene dendrogram and module colors. **(E)** The correlation between each module and the expression of immune metagenes.

To further excavate the prognosis of ovarian cancer immune microenvironment-related markers, we obtained the expression data for a total of 379 samples. A total of 15,268 transcripts with more than 75% TPM > 1 and median absolute deviation > median was selected from these samples. First, hierarchical clustering was used for cluster analysis of the samples, as shown in Figure 2A. There were some outlier samples. We screened the samples with a distance of more than 47,000 as outlier samples and finally obtained a total of 328 samples. Second, Pearson correlation coefficient was used to calculate the distance between each transcript. WGCNA was used to construct a weighted co-expression network. A soft threshold of 8 was selected to screen the co-expression modules. The research showed that the co-expression network conforms to the scale-free network; that is, the log(k) of the node with connectivity k was negatively correlated with the log(P(k) of the probability of the node, and the correlation coefficient was >0.8. To ensure that the network was scale-free, we select *β* = 8 (Figures 2B,C). Third, the expression matrix was transformed into an adjacency matrix, and then the adjacency matrix was transformed into a topological matrix. Based on the topology overlap matrix (TOM), we used the average-linkage hierarchical clustering method to cluster the genes. According to the standard of the hybrid dynamic shear tree, the minimum number of genes in each gene network module was set to 30. After determining the gene module by using the dynamic shearing method, we successively calculated the characteristic vector value (eigengenes) of each module and then performed cluster analysis on the module to merge the modules that were close to each other into new modules. Height = 0.25, deepSplit = 2, and minModuleSize = 30 were the set values. A total of 62 modules were obtained (Figure 2D). The gray module is the gene set that cannot be aggregated into other modules. The transcript statistics of each module are shown in Supplementary Table 4, from which 8,047 transcripts were assigned to 62 co-expression modules. We calculated the correlation between the feature vectors of the 62 modules and the immune metagenes, as shown in Figure 2E. The sienna3, yellow, antiquewhite4 and ivory modules have the highest correlations with the immune metagenes, with an average correlation >0.39. The number of transcripts in the four modules was 69, 378, 33, and 54, respectively, containing a total of 534 genes.

**FIGURE 3:**
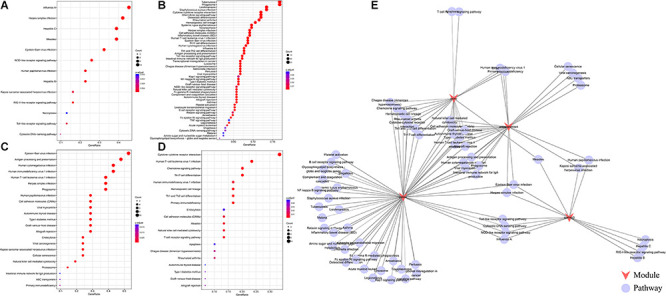
**(A)** The enrichment results of the sienna3module. The larger the circle is, the more module genes are containedin the pathway. The redder the color is, the more significant the gene was. **(B)** The pathways enriched by the yellow module.**(C)** The pathways enriched by the antiquewhite4 module. **(D)** The pathways enriched by the ivory module. **(D)** An interactive network of pathways enriched by the four modules. **(E)** An interactive network of pathways enriched by the four modules.

We further analyzed the function of genes in the four modules most related to immune metagenes. Among the four modules, the sienna3 module was enriched into 13 pathways. The yellow module was enriched into 54 pathways. The antiquewhite4 module was enriched into 23 pathways. The ivory module was enriched in 20 pathways. The relationship between the pathways enriched by these four modules was analyzed (Figure 3); There are 70 pathways enriched by the four modules, of which 31 are enriched by two or more modules, respectively. This result indicates that there are many intersections between the enriched pathways, of which eight are enriched by three modules at the same time (allograft rejection, autoimmune thyroid disease, cell adhesion molecules, Epstein-Barr virus infection, graft-vs.-host disease, herpes simplex infection, human T-cell leukemia virus 1 infection NK cell-mediated cytotoxicity, and type I diabetes mellitus). These pathways are closely related to immunity and cell adhesion.

To select genes associated with immune metagenes, we calculated the correlation between the gene and module and analyzed the correlation distribution of these genes as shown in Supplementary Figure 5. These correlation coefficients presented a bimodal distribution. With 0.72 as the critical point, we selected 248 genes with the maximum correlation coefficient >0.72.

### Differential Gene Analysis of Immune Differential Samples

Most of the immune metagenes are related to the prognosis, and the most significant type of immune metagenes such as ImmuneScore and STAT1 were selected. First, samples were divided into two groups, high ImmuneScore group and low ImmuneScore group, based on the average according to the ImmuneScore level. Then, the R software package DESeq2 was used to analyze the differentially expressed genes between the two groups of samples. In total, 219 differentially expressed genes were obtained, as shown in Supplementary Figure 6A, indicating that the up-regulated genes were significantly larger than the down-regulated genes and that up-regulated multiple genes was larger than the down-regulated multiple genes, in general. The expression profiles of these 219 genes are further visualized in Supplementary Figure 6B; there were obvious differences in the expression of differentially expressed genes in the high ImmuneScore group and low ImmuneScore group. Similarly, the samples were divided into two groups, the high STAT1 group and the low STAT1 group, based on the average according to the level of STAT1. Differentially expressed genes were screened by DESeq2, as shown in Supplementary Figures 6C,D. The differences in the STAT1 distribution results are similar to those in the ImmuneScore, and the expression levels were significantly higher for high-expression genes than in low-expression genes.

### Screening of Immune Microenvironment Genes With Prognostic Value

**FIGURE 4:**
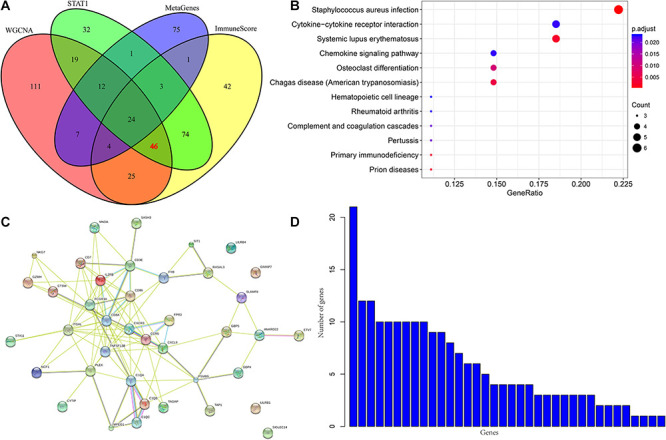
**(A)** Venn diagram. **(B)** Analysis results of KEGG enrichment. **(C)** Protein-protein interaction network. **(D)** Degree distribution of protein-protein interaction network.

To further analyze the co-expression relationship between genes with different immune scores and immune metagenes, we integrated 248 genes associated with the four most relevant metagenes modules, 219 genes with differential expression from ImmuneScore and 211 genes with differential expression from STAT1. We selected a total of 70 genes from all three, excluding 24 genes in 13 immune metagenes and resulting in 46 genes, as shown in Figure 4A. Next, we used the R software package clusterProfiler for KEGG enrichment analysis of these genes, and the selection threshold FDR < 0.05 is shown in Figure 4B. A total of 19 genes were enriched into 12 pathways, and most of these genes are related to immune diseases. The protein network interaction of these 46 genes were analyzed by using the R package STRINGdb. First, the 46 genes were mapped into the STRING database, and the network relationships among these genes were obtained as shown in Figure 4C. A total of 104 edges and 40 nodes were obtained. We analyzed the degree distribution of nodes in these networks as shown in Figure 4D. From this result, the degree of each node is higher, with an average degree of 5.7, indicating that these genes are closely related.

To screen genes with prognostic value in the immune microenvironment, we first analyzed the relationship between the expression of these 46 genes and prognosis using univariate survival analysis based on the prognostic information of the samples. A total of 14 genes were obtained by selecting *p* < 0.05 as the threshold, as shown in Table 1. The hazard ratio (HR) of these 14 genes was less than 1, and their high expression was related to good prognosis. Furthermore, we used clinical stages as a covariant to analyze the relationship between these genes and prognosis to exclude the influence of clinical stages and ultimately obtained 10 independent prognostic factors, as shown in Table 2.

**TABLE 1:** Genes with prognostic value.

**Genes**	***p*-value**	**HR**	**Low 95%CI**	**High 95%CI**
ENSG00000225492	0.00023	0.9584	0.936978	0.980312
ENSG00000168394	0.001941	0.995765	0.993095	0.998441
ENSG00000138755	0.002792	0.994868	0.991517	0.998229
ENSG00000240065	0.00507	0.995758	0.992802	0.998723
ENSG00000211753	0.007477	0.975419	0.957792	0.993371
ENSG00000162654	0.008348	0.991075	0.984494	0.997699
ENSG00000211772	0.010813	0.986706	0.976604	0.996913
ENSG00000256262	0.013449	0.951503	0.914724	0.989761
ENSG00000010030	0.019584	0.978497	0.9608	0.996521
ENSG00000198851	0.02893	0.982099	0.966311	0.998146
ENSG00000277734	0.030677	0.990207	0.98141	0.999084
ENSG00000154451	0.031368	0.974711	0.95224	0.997713
ENSG00000152766	0.037442	0.961857	0.927262	0.997742
ENSG00000206337	0.048843	0.994756	0.989566	0.999973

**TABLE 2:** Stages were introduced as covariates to obtain significant prognostic genes.

**Genes**	***p*-value**	**HR**	**Low 95%CI**	**High 95%CI**	**Entrezid**	**Symbol**
ENSG00000138755	0.00849	0.995426	0.992033	0.99883	4,283	CXCL9
ENSG00000010030	0.04061	0.981223	0.963579	0.99919	51,513	ETV7
ENSG00000162654	0.022077	0.992104	0.985392	0.998861	115,361	GBP4
ENSG00000211772	0.02085	0.987918	0.977784	0.998157	28638	TRBC2
ENSG00000225492	0.00091	0.962018	0.940256	0.984283	400,759	GBP1P1
ENSG00000198851	0.048451	0.983727	0.967827	0.999888	916	CD3E
ENSG00000256262	0.026533	0.956429	0.919515	0.994825	100,131,733	USP30-AS1
ENSG00000211753	0.013667	0.977354	0.959721	0.995311	28,559	TRBV28
ENSG00000168394	0.005226	0.996155	0.993465	0.998852	6,890	TAP1
ENSG00000240065	0.009633	0.996091	0.993141	0.999049	5,698	PSMB9

According to the expression levels of these 10 prognostic genes (CXCL9, ETV7, GBP4, TRBC2, GBP1P1, CD3E, USP30-AS1, TRBV28, TAP1, and PSMB9), we divided the samples into two groups according to the median expression levels. The prognostic differences between the high-expression group and the low-expression group were analyzed. As shown in Supplementary Figures 7, 9 of the 10 genes with a high-expression prognosis were significantly better than the low-expression prognosis. There was a significant trend in the TRBV28 gene, but it was not obvious. This may be because the 5-year survival rate is inseparable, but the prognosis is obviously different after 5 years.

**FIGURE 5:**
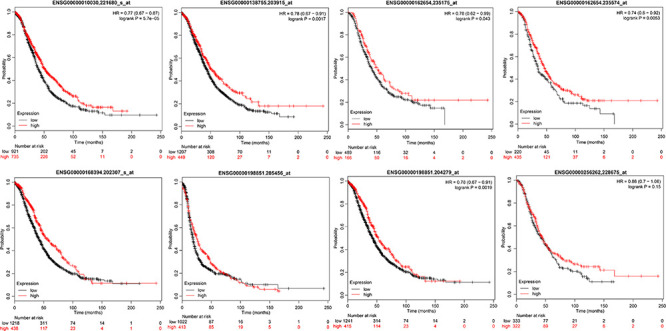
The prognostic KM curve of the seven genes in the KMplot platform.

To verify the relationship between these 10 genes and prognosis, we used the online tool KMplot to analyze the relationship between these 10 genes and overall survival in ovarian cancer. We retrieved 6 genes from the KMplot platform. The KM curves of these 6 genes (two of which have two probes) are shown in Figure 5, and six genes were characterized by a high expression of prognosis as being good. Five of these genes (ETV7, GBP4, CXCL9, CD3E, and TAP1) are significantly correlated with prognosis, which is consistent with our analysis.

### Evaluation of the Prognosis of Ovarian Cancer and Hub Genes by IHC

**FIGURE 6:**
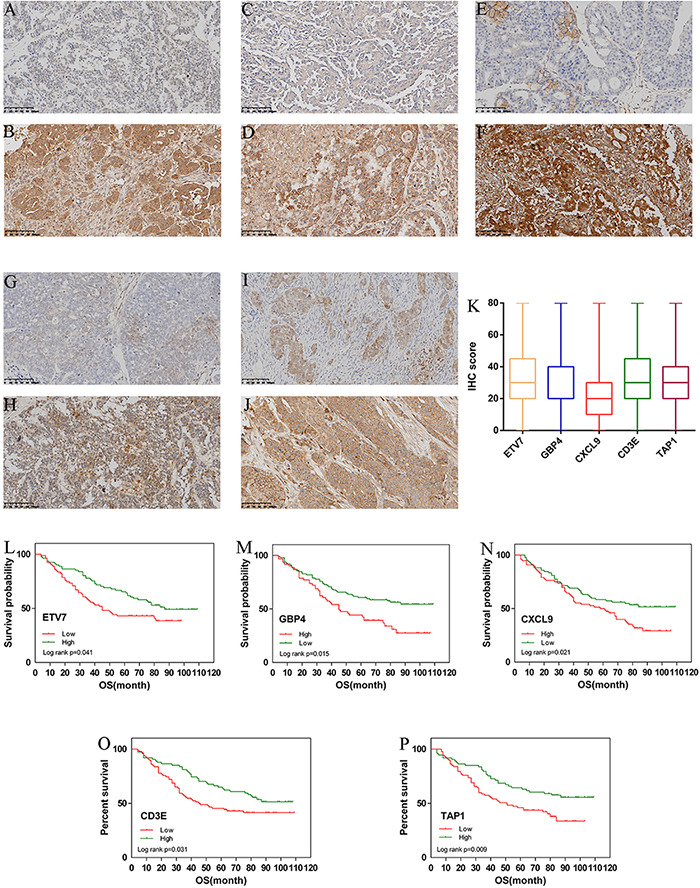
Immunohistochemistry for ETV7, GBP4, CXCL9, CD3E, and TAP1. Samples of ovarian cancer (*N* = 168). Ovarian cancer sample of weak and strong immunostaining score for ETV7 **(A,B)**, GBP4 **(C,D)**, CXCL9 **(E,F)**, CD3E **(G,H)**, and TAP1 **(I,J)**, respectively. Expression of each gene is depicted in **(K)** slides. (X 100). Overall survival (OS) curves for ovarian cancer (*N* = 168) according to ETV7 **(L)**, GBP4 **(M)**, CXCL9 **(N)**, CD3E **(O)**, and TAP1 **(P)** gene expression status (low or high). Geneexpression status was divided according to their median values.

From January 2010 and January 2015, 168 human ovarian tissue samples which had accompanying follow-up information. Supplementary Table 8 summarizes the characteristics of all patients, including age, disease stage, and tumor grade. We selected the five hub genes to evaluate gene expression values by IHC. The expression of ETV7 (33.13 ± 1.65), GBP4 (28.48 ± 1.48), CXCL9 (23.30 ± 1.30), CD3E (36.52 ± 1.59), and TAP1 (29.94 ± 1.37) are shown in Figures 6A–K. The correlation between expression of these genes and ovarian cancer prognosis is shown in Figures 6L–P. These data reveal that high expression of ETV7 (OS, HR = 1.540, 95% CI 1.023–2.390, *p* = 0.041), GBP4 (OS, HR = 1.834, 95% CI 1.242–3.055, *p* = 0.004), CXCL9 (OS, HR = 1.613, 95% CI 1.080 –2.471, *p* = 0.021), CD3E (OS, HR = 1.590, 95% CI 1.049 –2.459, *p* = 0.031), and TAP1 (OS, HR = 1.766, 95% CI 1.163 –2.723, *p* = 0.009) are associated with better prognosis in patients with ovarian cancer.

## Discussion

Ovarian cancer is the most common cause of death from gynecologic malignancy (Torre et al., 2015). Epithelial ovarian cancer (EOC) is the most common ovarian tumor with a lack of specific clinical symptoms at early stage, 75% of patients were diagnosed with advanced tumors (FIGO III/IV), and the standard of treatment was complete resection of all visible tumor lesions and platinum-based chemotherapy (Ferlay et al., 2015). Although most patients with advanced ovarian cancer respond to standard ovarian cancer therapeutic approaches, 70% of patients will eventually relapse and develop chemotherapy resistance (Hennessy et al., 2009). Therefore, more effective prognostic and therapeutic strategies to reduce the mortality rate of ovarian cancer are being actively explored. Stromal cells, extracellular matrix and exosomes comprise the tumor microenvironment. Intrinsic genes of tumor cells, especially master transcription factors, determine the occurrence, development and evolution of ovarian cancer, but the surrounding microenvironment interacts with tumor cells through secretory interactions, providing an impetus for the invasion and metastasis of tumor cells (Pietras and Ostman, 2010). In recent years, the tumor microenvironment has gradually been considered to play an important role in ovarian cancer metastasis and may become a potential biomarker for the diagnosis and treatment of ovarian cancer patients (Luo et al., 2016). To fully understand the biological behavior of ovarian cancer, it is necessary to consider the environment in which ovarian cancer cells exist and how they are manipulated by their surroundings to promote malignant phenotypes.

In recent years, with the development of sequencing technology, as well as public databases such as TCGA and Gene Expression Omnibus (GEO) database, a large number of studies have been conducted on human cancer gene expression. In ovarian cancer, Men et al. (2018) performed a genome-wide analysis of gene expression profiling in the TCGA and developed an 11 gene expression signature-based risk score that can predict a patient’s survival. In another study that used robust Bayesian network modeling, 13 hub genes including ARID1A, C19orf53, CSKN2A1, and COL5A2 signature with a prognostic function in ovarian cancer was established (Zhang et al., 2014; Guo et al., 2020). However, most studies focused on oncogene panels of ovarian cancer.

In present study, we performed a multistep bioinformatics analysis using data from the TCGA database and identified a list of tumor microenvironment-related genes that may contribute to ovarian cancer overall survival. We first used RNA-Seq data of ovarian cancer in the TCGA (379 samples) to systematically analyze the relationship between ovarian cancer and immune metagenes; we found that the expression of immune metagenes was closely related to the immune components in the tumor microenvironment. Next, the expression levels of immune metagenes in different stages were analyzed, and different stages had significant prognostic differences (Figure 2). Third, by analyzing the relationship between these immune metagenes and BRCA1 and BRCA2 mutations, the expression of immune metagenes was found to be related to only BRCA1 mutations. Finally, we screened 10 genes related to immunity and prognosis in ovarian cancer according to the expression of immune metagenes. By cross validation with KMplot, an independent cohort of 1,816 ovarian patients, we identified 5 tumor microenvironment-related genes that showed a significant correlation between gene expression and prognosis. Our results may provide new insights into the underlying biological mechanisms driving the tumorigenesis of ovarian cancer.

This study identified tumor microenvironment-related genes, including monokine induced by gamma interferon (MIG or CXCL9), E26 transformation-specific variant 7 (ETV7), guanylate binding protein 4 (GBP4), and CD3 epsilon chain (CD3E). In agreement with a previous study, we found that these genes were differentially expressed in a variety of human tumors and correlated with survival time. For example, CXCL9 is located on human chromosome 4 and is induced by IFN-γ but not by IFN-α/β. CXCL9 predominantly mediates lymphocytic infiltration to the focal sites and suppresses tumor growth (Gorbachev et al., 2007). CXCL9 can predict survival and is regulated by cyclooxygenase inhibition in advanced serous ovarian cancer (Bronger et al., 2016). Wu et al. used the KM method as well as Cox’s univariate and multivariate hazard regression models and found that the higher the CXCL9 expression is, the higher the overall survival rate for colorectal carcinoma patients (Wu et al., 2016). In addition, plasma CXCL9 has been found to predict the survival of patients with advanced pancreatic ductal adenocarcinoma receiving chemotherapy, potentially improving treatment outcomes (Qian et al., 2019). In cervical carcinoma, low expression of CD3E was correlated with poor disease-specific and disease-free survival, and high CD3E expression was correlated with improved disease-specific survival (Punt et al., 2015). Moreover, this gene was also considered as a hub gene in head and neck squamous cell carcinoma (Upreti et al., 2016). A high expression of GBP4 was correlated with favorable overall survival in skin (cutaneous) melanoma patients followed for over 30 years (Wang et al., 2018). Therefore, these gene-associated tumor microenvironments may serve as important roles in the pathogenesis of ovarian cancer.

However, our study may have some disadvantages. First, there is a lack of experimental research that can explain the biological significance and molecular mechanism of immune microenvironment genes in ovarian cancer. Second, a small portion of the results are not statistically significant, but there is a trend difference, which may be due to the limited sample size. Third, the prognostic value of these immune microenvironment genes needs to be validated from a large independent cohort before they can be applied to clinical practice. Moreover, the microenvironment gene also significantly associated with the prognosis of other histology types ovarian cancer has been rarely studied in present research. According to histological and pathological morphological differences, ovarian cancer can be divided into various types: serous carcinoma, mucinous carcinoma, endometrioid carcinoma, clear cell carcinoma and other types of tumors. Different types of ovarian cancer have obvious clinical pathological differences and molecular differences (Tone et al., 2008). However, since more than 70% of ovarian epithelial cancer are serous types, there are no enough samples of other types in the dataset from TCGA for effective analysis. In further study, we will pay more attention to the prognosis between the microenvironment genes and other types of ovarian cancer.

## Conclusion

In conclusion, through a comprehensive analysis of the data of ovarian cancer patients, we found a group of immune microenvironment genes that can be used as potential biomarkers to predict the prognosis of ovarian cancer patients. This study provides a new understanding of the potential relationship between the tumor microenvironment and ovarian cancer prognosis and provides a new molecular target for the development of more effective treatment methods for ovarian cancer. This study will help refine and personalize treatment.

## Data Availability Statement

The original contributions presented in the study are included in the article/Supplementary Material, further inquiries can be directed to the corresponding author/s.

## Ethics Statement

The studies involving human participants were reviewed and approved by the Beijing Chao-Yang Hospital. The patients/participants provided their written informed consent to participate in this study.

## Author Contributions

XH: study design, manuscript writing, and data analysis. HS: data analysis, data collection, manuscript writing, and resources. SL: study design, resources, and data analysis. SW: funding and resources. BL and HB: resources. All authors have read, edited and approved of the final version of the manuscript.

## Conflict of Interest

The authors declare that the research was conducted in the absence of any commercial or financial relationships that could be construed as a potential conflict of interest.

## Footnotes

1 http://kmplot.com

2 http://cancergenome.nih.gov

3 https://cistrome.shinyapps.io/timer/
