# Supplementary material for: Identification of a Prognostic Signature for Ovarian Cancer Based on the Microenvironment Genes
Source: Front Genet. 2021 May 13;12:680413. doi: 10.3389/fgene.2021.680413 (PMC8155613; doi:10.3389/fgene.2021.680413)
Supplement: Supplementary file 1 [file Data_Sheet_1.docx]

**Supplementary S1:** The correlation between immune metagenes and scores related to other types of immunity. A: Correlation analysis between immune metagenes. B: Correlation between immune metagenes and immune cells. C: The correlation between immune metagenes and immune and stromal scores.


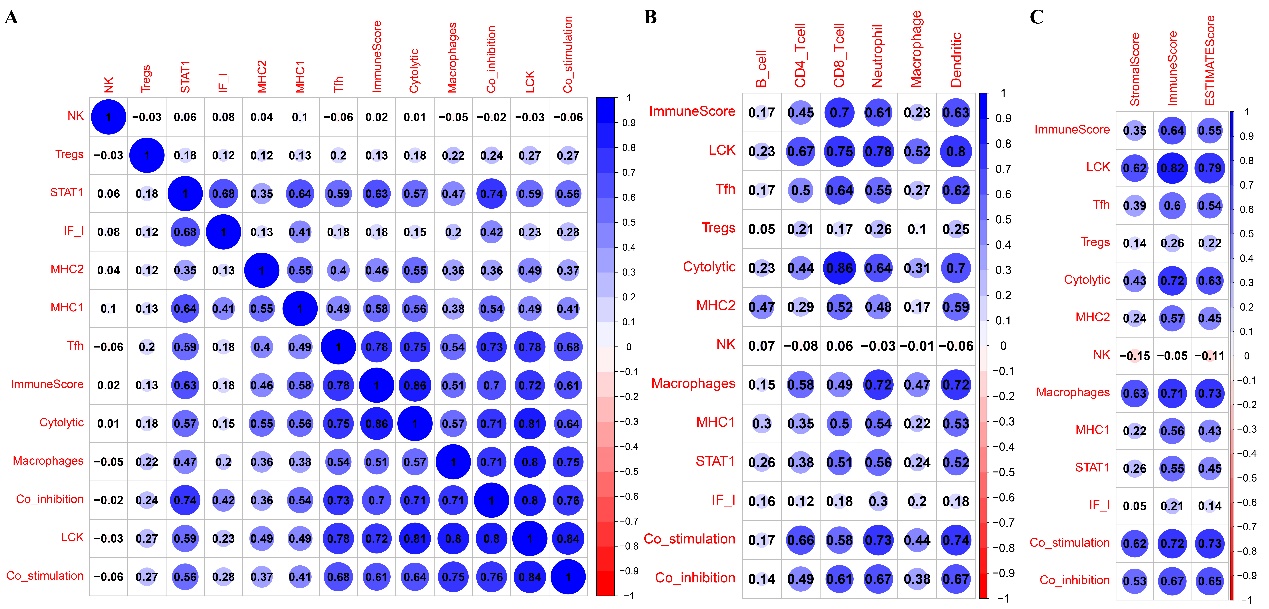


Note: NK (natural killer cells), Tregs (regulatory T cells), IF_I (interferon-inducible genes), MHC2 (major histocompatibility complex class II antigen), Tfh (follicular helper T cells) and STAT1 (signal transducer and activator of transcription 1), Cytolytic: immune cytolytic activity, Macrophages: tumor-associated macrophages, co_inhibition: co-inhibitory receptors (T cells to negatively), LCK: The expression of lymphocyte-specific kinase metagene, co_stimulation: co-stimulatory receptors (T cells to positively).

**Supplementary S2:** A-M: The expression distribution of 13 types of immune metagenes in Stage II, Stage III and Stage IV. N: The Kaplan-Meier (KM) curve of prognostic differences between the four stages.


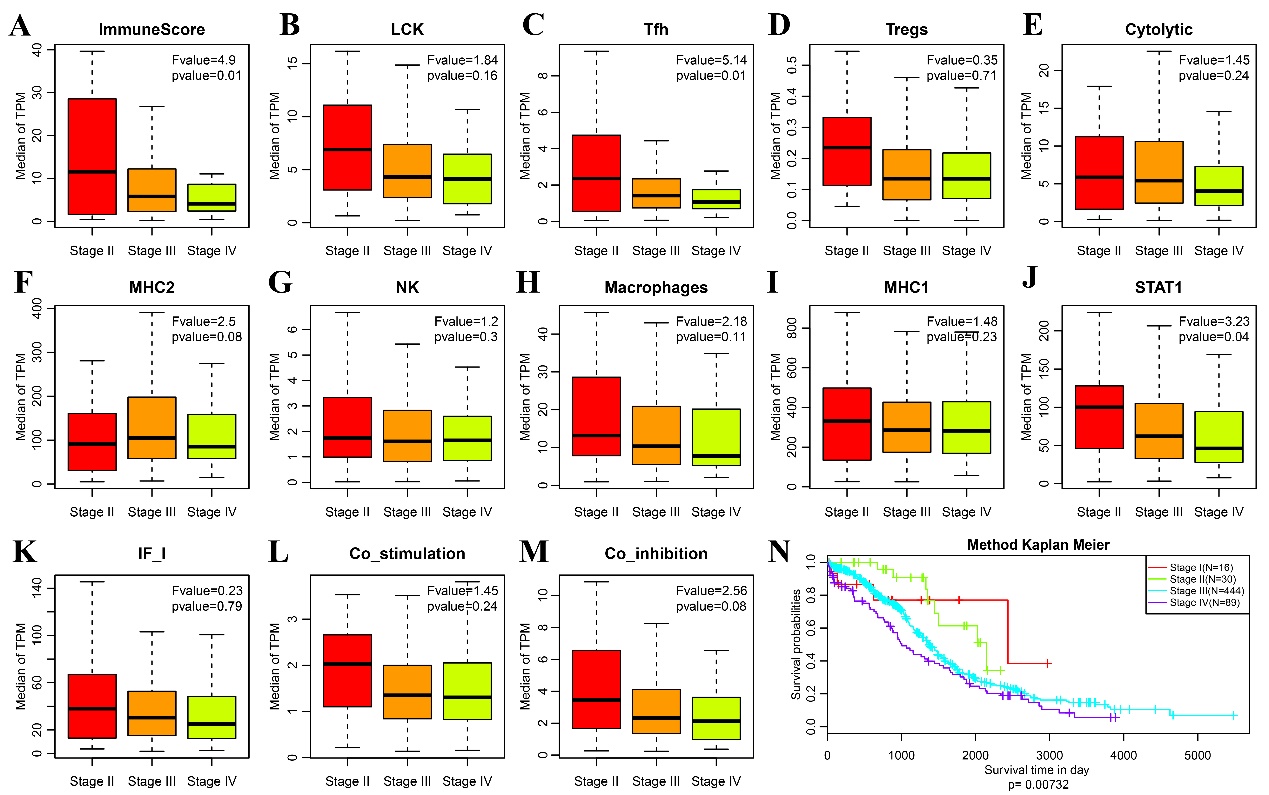


**Supplementary S3:** A-M: The differential expression of immune metagenes between the BRCA1 mutation group and the wild-type group. N-Z: The differential expression of immune metagenes between the BRCA2 mutation group and the wild-type group. Red represents the mutant group, and green represents the wild-type. The Wilcoxon rank sum test was used for the analysis.


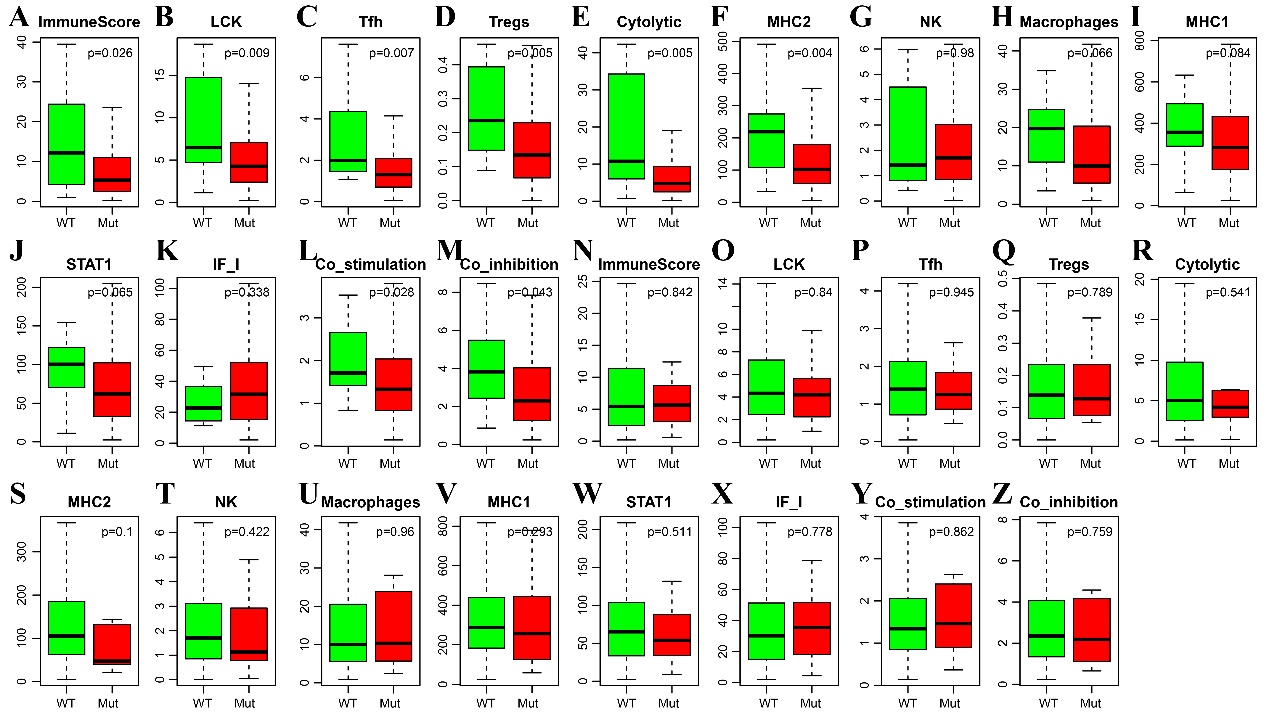


**Supplementary S4.** Count the number of genes corresponding to each module

| **Modules** | **Genes** | **Modules** | **Genes** | **Modules** | **Genes** |
| --- | --- | --- | --- | --- | --- |
| antiquewhite4 | 33 | lightsteelblue1 | 58 | yellow | 378 |
| bisque4 | 48 | lightyellow | 101 | yellowgreen | 68 |
| black | 236 | magenta | 159 | lightpink4 | 42 |
| blue | 677 | maroon | 43 | white | 82 |
| brown | 511 | mediumpurple3 | 60 |  |  |
| brown4 | 49 | midnightblue | 111 |  |  |
| coral1 | 33 | navajowhite2 | 43 |  |  |
| cyan | 112 | orange | 82 |  |  |
| darkgreen | 91 | orangered4 | 60 |  |  |
| darkgrey | 83 | paleturquoise | 72 |  |  |
| darkmagenta | 70 | palevioletred3 | 43 |  |  |
| darkolivegreen | 70 | pink | 159 |  |  |
| darkorange | 82 | plum1 | 64 |  |  |
| darkorange2 | 50 | plum2 | 47 |  |  |
| darkred | 100 | purple | 148 |  |  |
| darkseagreen4 | 37 | red | 249 |  |  |
| darkslateblue | 48 | royalblue | 101 |  |  |
| darkturquoise | 87 | saddlebrown | 81 |  |  |
| floralwhite | 50 | salmon | 115 |  |  |
| green | 364 | salmon4 | 44 |  |  |
| greenyellow | 142 | sienna3 | 69 |  |  |
| grey | 7221 | skyblue | 81 |  |  |
| grey60 | 105 | skyblue3 | 65 |  |  |
| honeydew1 | 42 | steelblue | 76 |  |  |
| ivory | 54 | tan | 137 |  |  |
| lavenderblush3 | 42 | thistle1 | 44 |  |  |
| lightcyan | 105 | thistle2 | 45 |  |  |
| lightcyan1 | 57 | turquoise | 1568 |  |  |
| lightgreen | 103 | violet | 71 |  |  |

**Supplementary S5.** Distribution of correlation coefficients between 534 genes and four modules.


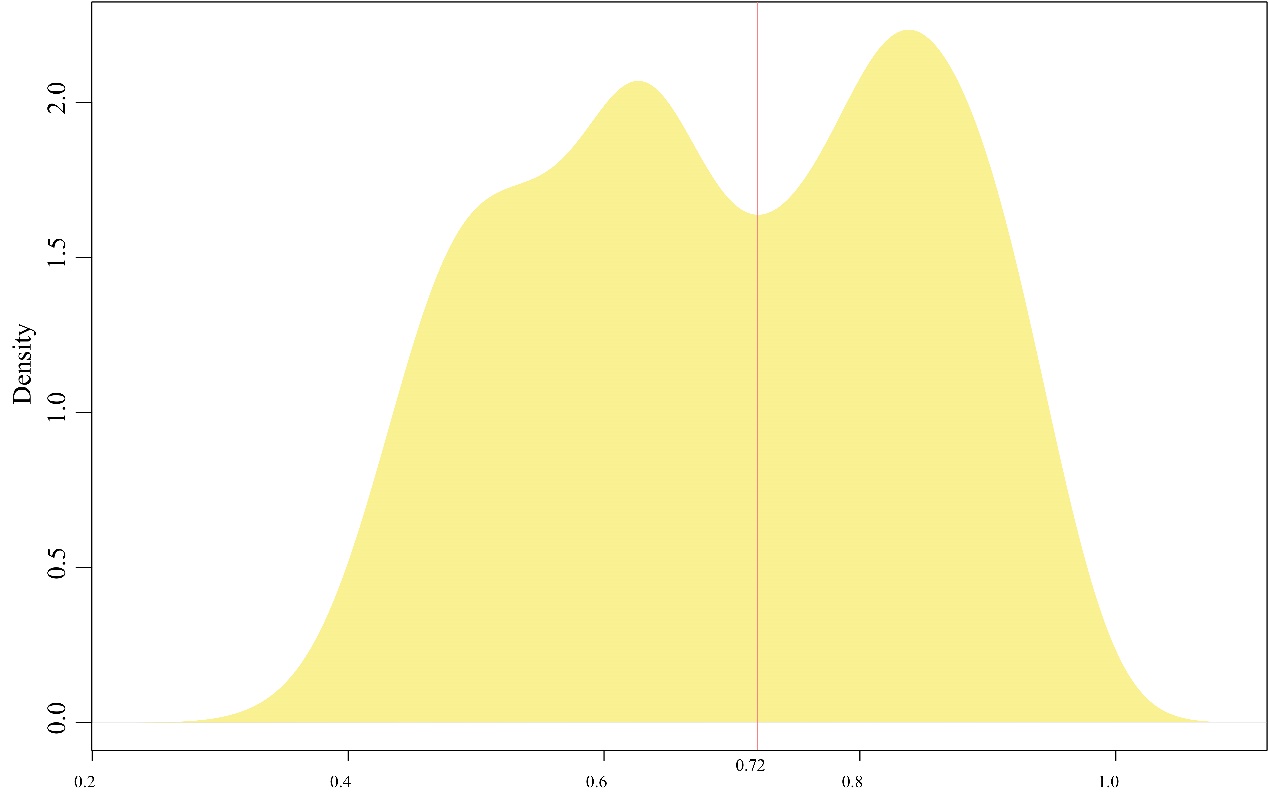


**Supplementary S6.** A: Clustering heat map of different genes grouped by the expression from ImmuneScore. B: Volcano map of differentially expressed genes grouped by the expression from ImmuneScore. C: Clustering heat map of different genes grouped by expression from STAT1. D: Volcano map of differentially expressed genes grouped by the expression from STAT1.


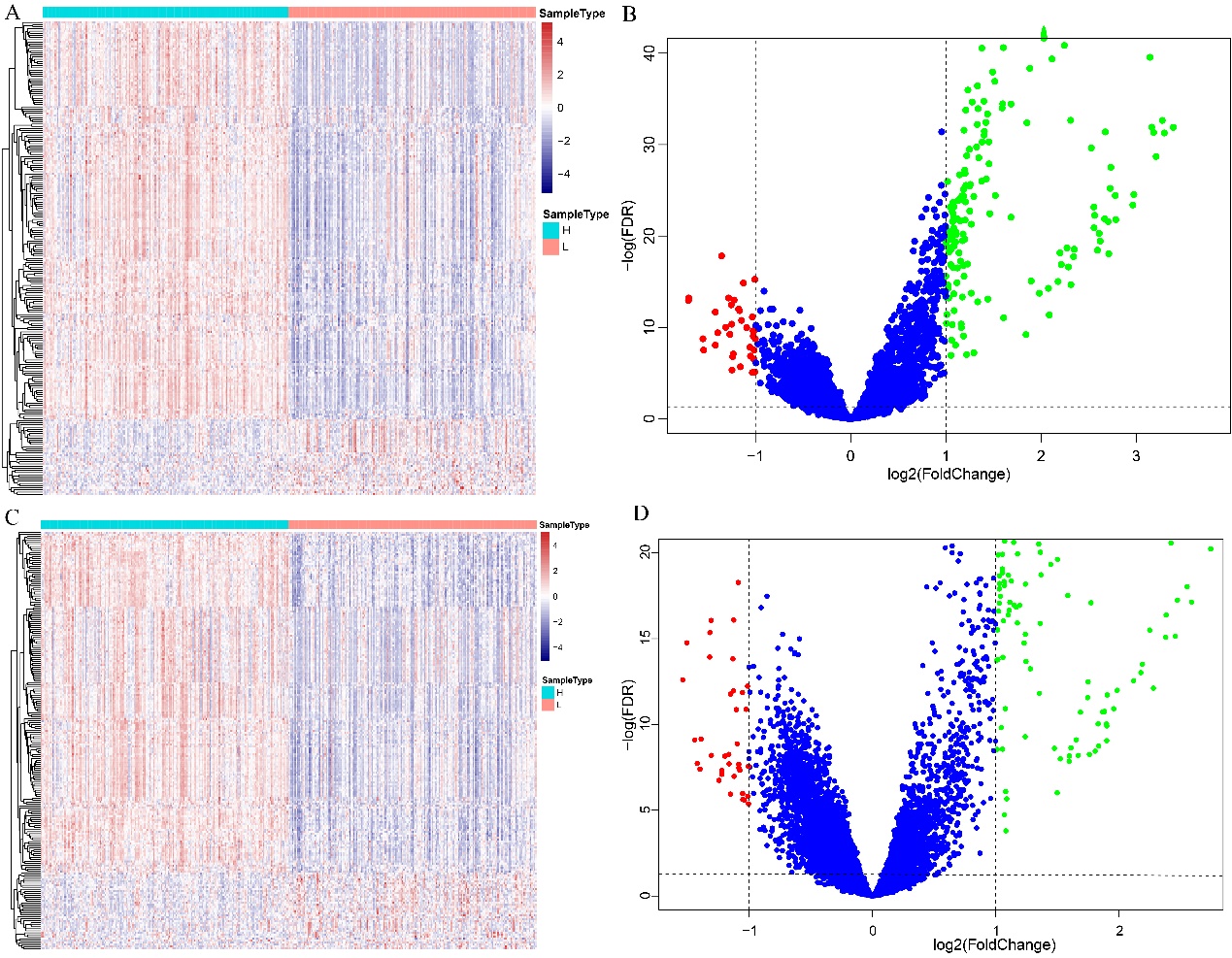


**Supplementary S7.** The KM curves of 10 immune-related prognostic genes.


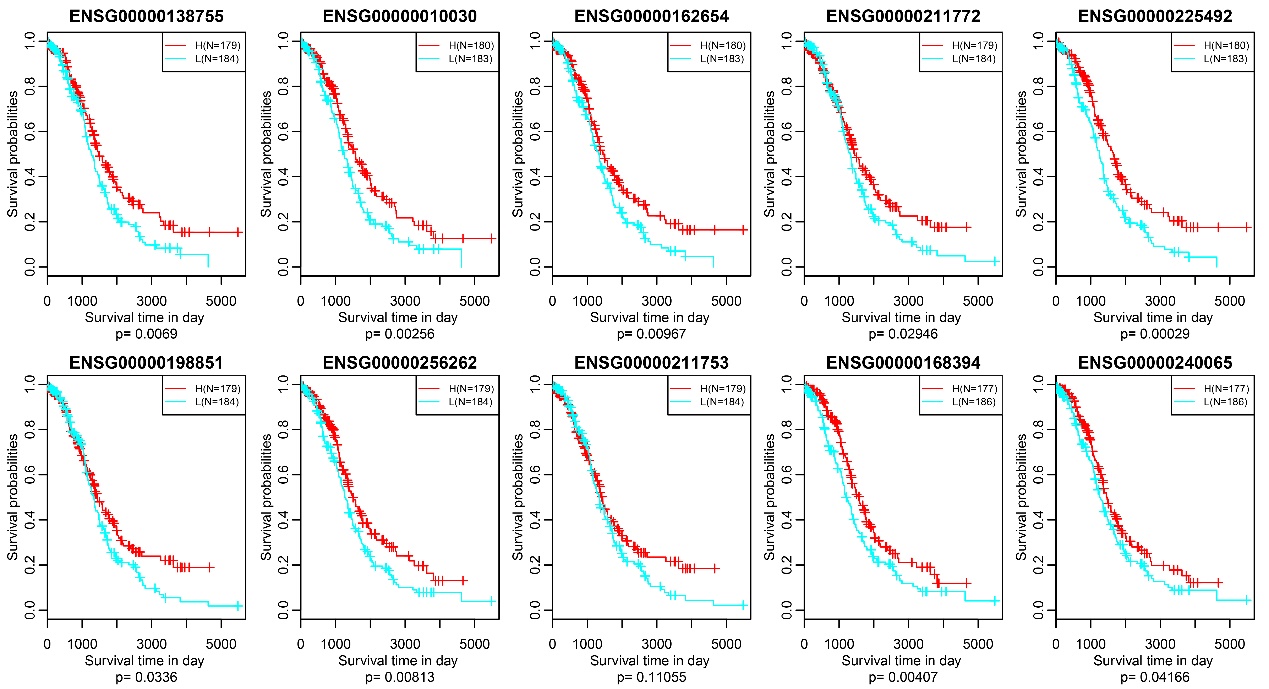


**Supplementary S8.** Characteristics of ovarian cancer patients

| **Characteristic** | **No.** | **%** |
| --- | --- | --- |
| **Age (y), median (range)** | 54(28-76) | 100% |
| **Stage** |  |  |
| I–II | 42 | 25.0% |
| III | 104 | 61.9% |
| IV | 22 | 13.1% |
| **Histological subtype** |  |  |
| Serous | 142 | 84.5% |
| Endometroid | 8 | 4.8% |
| Clear cell | 12 | 7.1% |
| Mullerian ductal | 6 | 3.6% |
| **Differentiation grade** |  |  |
| G1 | 40 | 23.8% |
| G2 | 45 | 26.8% |
| G3 | 77 | 45.8% |
| Unknown | 6 | 3.6% |
| **Residual disease** |  |  |
| optimal | 114 | 67.8% |
| suboptimal | 28 | 16.7% |
| unknown | 26 | 15.5% |
